# Facilitating sexual and reproductive health services for adolescent girls in the COVID-19 era: An urgent public health priority

**DOI:** 10.4102/phcfm.v14i1.3357

**Published:** 2022-01-25

**Authors:** Grant Murewanhema, Godfrey Musuka, Tafadzwa Dzinamarira

**Affiliations:** 1 Department of Primary Health Care Sciences, Faculty of Medicine, University of Zimbabwe, Harare, Zimbabwe; 2 ICAP at Columbia University, Harare, Zimbabwe; 3 Department of Health Systems and Public Health, Faculty of Health Sciences, University of Pretoria, Pretoria, South Africa

Almost 18 months into the coronavirus disease 2019 (COVID-19) pandemic, sexual and reproductive health (SRH) concerns for adolescent girls in Zimbabwe remain unresolved.^1^ The control measures for COVID-19 pandemic such as lockdowns and closure of non-essential health services resulted in disruption of SRH services for this critical population. The disruptions have resulted in failure to access family planning (FP) clinics, emergency contraception, abortion facilities in cases of unintended pregnancies, treatment for sexually transmitted infections (STIs) and attendance for sexual and gender-based violence (SGBV) victims, and disruptions in human papilloma virus (HPV) vaccination programmes.^1^ This correspondence is, therefore, a call for prioritisation of restoration and maintenance of robust SRH services for adolescent girls in Zimbabwe to militate against further negative impact on SRH outcomes in this population.

At the beginning of the pandemic, it was projected that there would be an increase in maternal deaths in sub-Saharan Africa because of disruptions in SRH services.^2,3^ In Zimbabwe, this has been worsened by increasing healthcare worker (HCW) attrition, HCW fear of contracting COVID-19, lack of personal protective equipment (PPE) and failing to timeously access healthcare services. The public transport crisis in the country has continued to worsen because of restrictions in public transport provision, poor roads and digital networks. Based on the three-delays conceptual framework, first-stage delays are occurring because of late decisions in seeking care at primary facilities, second-stage delays as a result of logistical challenges in getting patients to higher facilities, and third-stage delays at higher level facilities owing to lack of human resources, PPE, medicines and other sundries.

Prolonged closure of schools resulted in young girls spending more time in the community and indulging in early premarital sexual activities.^4,5^ Thus, an increased incidence of teenage pregnancies has been widely reported. Many teenagers who fall pregnant never go back to school, and they end up in abusive relationships or informal jobs, including commercial sex work and cross-border trading. This may expose them to multiple sexual partners and frequent unprotected intercourse, which are high-risk behaviours for SRH.^6^

We, therefore, urge the government and relevant public health stakeholders to prioritise restoring and maintaining robust SRH services for adolescent girls. Amongst other things, the actions should include restoring FP services, HPV vaccination programmes, rape and SGBV clinics, to ensure timely access to treatment and prevention services. In addition, re-opening of schools and appropriate SRH education for this population remain indispensable components of adolescent SRH.
